# Pulmonary, Central Nervous System, and Systemic Paradoxical Cardioembolic Events Secondary to Staphylococcus aureus Tricuspid Valve Infective Endocarditis Without Intracardiac Shunt

**DOI:** 10.7759/cureus.97827

**Published:** 2025-11-26

**Authors:** Richard Lyon, Ihab Abdlaziz

**Affiliations:** 1 Critical Care Medicine, Lewisham and Greenwich NHS Trust, London, GBR; 2 Anesthesia and Critical Care Medicine, Lewisham and Greenwich NHS Trust, London, GBR

**Keywords:** brain abscess, infective endocarditis, paradoxical embolism, pulmonary embolism, septic shock, staphylococcus aureus, tricuspid valve

## Abstract

Infective endocarditis (IE) is an uncommon but potentially fatal cause of systemic infection that may lead to embolic complications in various organs. While left-sided IE commonly causes systemic emboli, right-sided IE typically results in septic pulmonary emboli due to embolization of infected material into the pulmonary circulation.

This report describes an unusual case of right-sided tricuspid valve IE caused by *Staphylococcus aureus*, which resulted in simultaneous pulmonary, cerebral, and peripheral embolic events despite the absence of an identifiable intracardiac or intrapulmonary shunt. A previously healthy middle-aged man presented with fever, confusion, and hemodynamic instability and was found to have extensive septic emboli affecting the lungs, brain, and lower extremities. Diagnostic imaging confirmed large vegetations on the tricuspid valve, while echocardiography ruled out a patent foramen ovale or left-sided involvement. The patient was treated medically with targeted intravenous antibiotics, renal replacement therapy, and vasopressor support, leading to clinical improvement. However, he developed irreversible peripheral ischemia requiring partial amputation and residual renal impairment. This case underscores that right-sided IE can, in rare circumstances, produce paradoxical systemic emboli through mechanisms that remain poorly understood. Awareness of this possibility and early multidisciplinary management are crucial to reducing complications and improving survival.

## Introduction

Infective endocarditis (IE) is an uncommon but potentially devastating cause of systemic sepsis, with an estimated incidence of three to nine cases per 100,000 persons annually. Embolization from valvular vegetations can cause significant morbidity and mortality. Left-sided IE is typically associated with systemic embolic events, while right-sided disease more often results in septic pulmonary emboli. Systemic emboli from right-sided lesions are termed paradoxical emboli. This phenomenon is rare and usually attributed to an intracardiac shunt such as a patent foramen ovale.

This study is unique because systemic, cerebral, and pulmonary emboli occurred simultaneously without any detectable intracardiac or intrapulmonary shunt, despite extensive imaging including transthoracic echocardiography (TTE), transesophageal echocardiography (TEE), and CT pulmonary angiography. Existing literature describes only scattered similar cases.

Another essential differential diagnosis in embolic presentations is marantic (nonbacterial thrombotic) endocarditis (NBTE), which produces sterile vegetations and systemic emboli. NBTE, commonly associated with malignancy, autoimmune disease, or hypercoagulable states, must be considered during early evaluation. Its clinical overlap with IE has been highlighted in recent reviews.

This case is further notable due to the involvement of *Staphylococcus aureus*, an organism with unique virulence factors, including coagulase production, adhesins, cytotoxins, endothelial invasion, and strong biofilm formation, that predispose it to atypical metastatic manifestations. Finally, the patient’s presentation highlights the role of structural inequities and social determinants of health, including limited access to dental care and delayed treatment of oral infections, which contribute to increased cardiovascular infectious risk.

Here, we present a case of *Staphylococcus aureus *tricuspid valve infective endocarditis complicated by pulmonary, cerebral, and peripheral embolic events in the absence of a demonstrable intracardiac shunt or left-sided involvement.

## Case presentation

A previously healthy male in his 50s presented with a four-day history of fever, confusion, postural instability, and mild headache. His past medical history included only hypertension. He reported a recent episode of gingivitis two weeks prior, which resolved spontaneously without antibiotic therapy. He denied intravenous drug use, recent invasive procedures, or high-risk behaviors.

On arrival, he was hypotensive (79/56 mmHg), tachycardic with atrial fibrillation (rate 170 beats per minute), tachypneic (37 breaths per minute), and febrile, with mild confusion. There were no focal neurological deficits. He was admitted to the intensive care unit for septic shock and multiorgan dysfunction. He initially required vasopressor infusions via central venous access; however, his condition deteriorated, requiring intubation and mechanical ventilation alongside renal replacement therapy.

**Figure 1:**
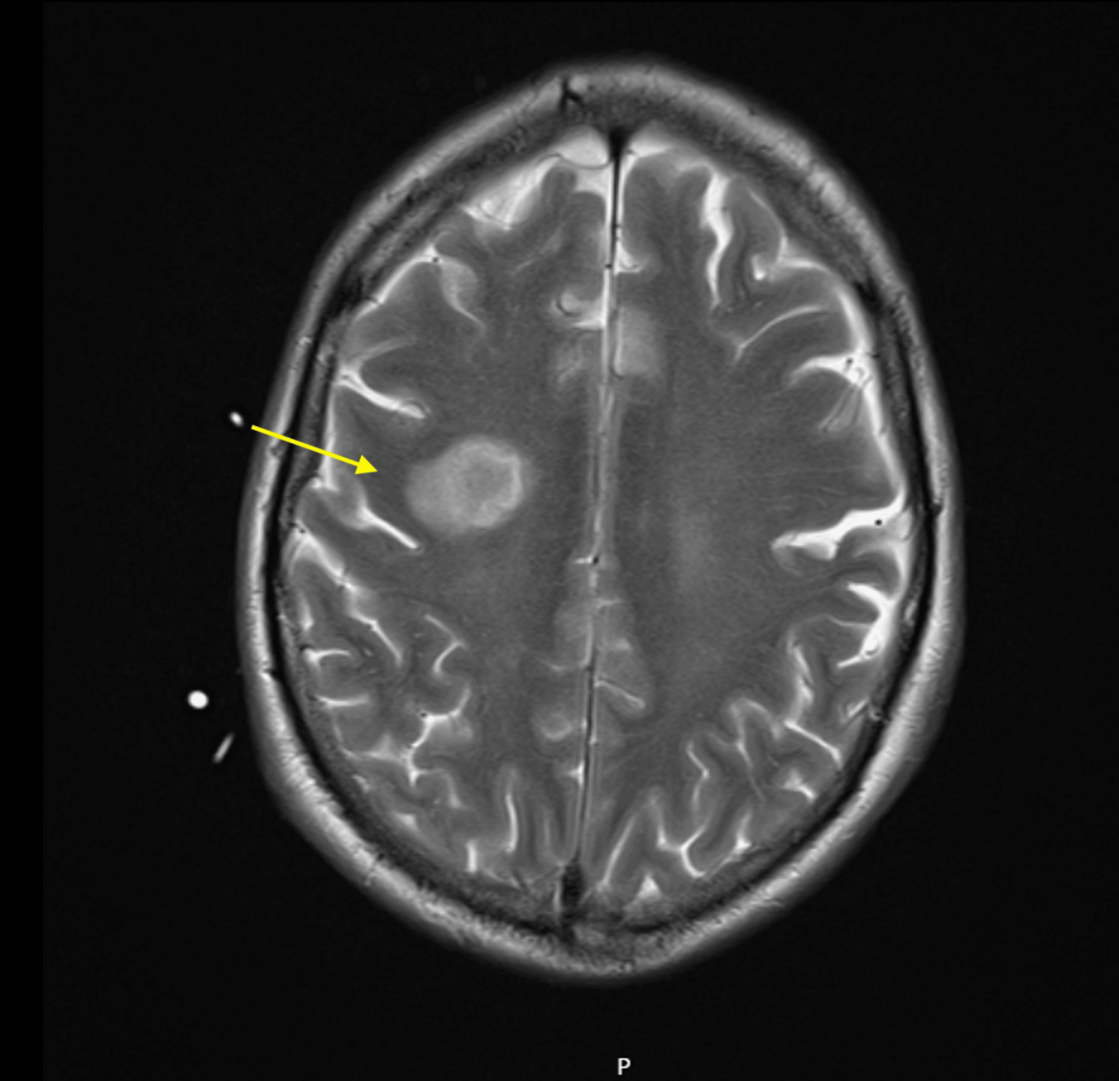
T2-weighted post-contrast MRI brain demonstrating a right frontal lobe septic embolus and abscess formation. The arrow annotation highlights the area of high T2 signal with early ring enhancement typical of cerebral abscess.

Laboratory investigations revealed markedly elevated inflammatory markers (CRP: 416 mg/L, WCC: 23×10^9^/L), acute kidney injury (creatinine: 463 μmol/L), and thrombocytopenia (platelets: 64×10^9^/L) (Table 1). Blood and cerebrospinal fluid cultures both grew *Staphylococcus aureus*. CT imaging demonstrated bilateral pulmonary abscesses and a right frontal lobe hypodensity, with MRI confirming a 20x14x17 mm abscess secondary to septic embolus (Figure 1). The patient was initially treated broadly, and antibiotics were selected based on CNS penetration while the source was identified. Antibiotics were de-escalated to specific treatment for IE rather than a primary meningitis, on microbiological advice. The cranial imaging findings were discussed with the neurosurgical service; however, the decision was made to proceed with medical management in the first instance.

**Table 1:** Serum laboratory findings on admission.

Parameters	Patient value	Normal range
C-reactive protein (CRP)	416 mg/L	<5 mg/L
White cell count (WCC)	23×10⁹/L	4-11×10⁹/L
Creatinine	463 μmol/L	60-110 μmol/L
Platelets	64×10⁹/L	150-400×10⁹/L

**Figure 2:**
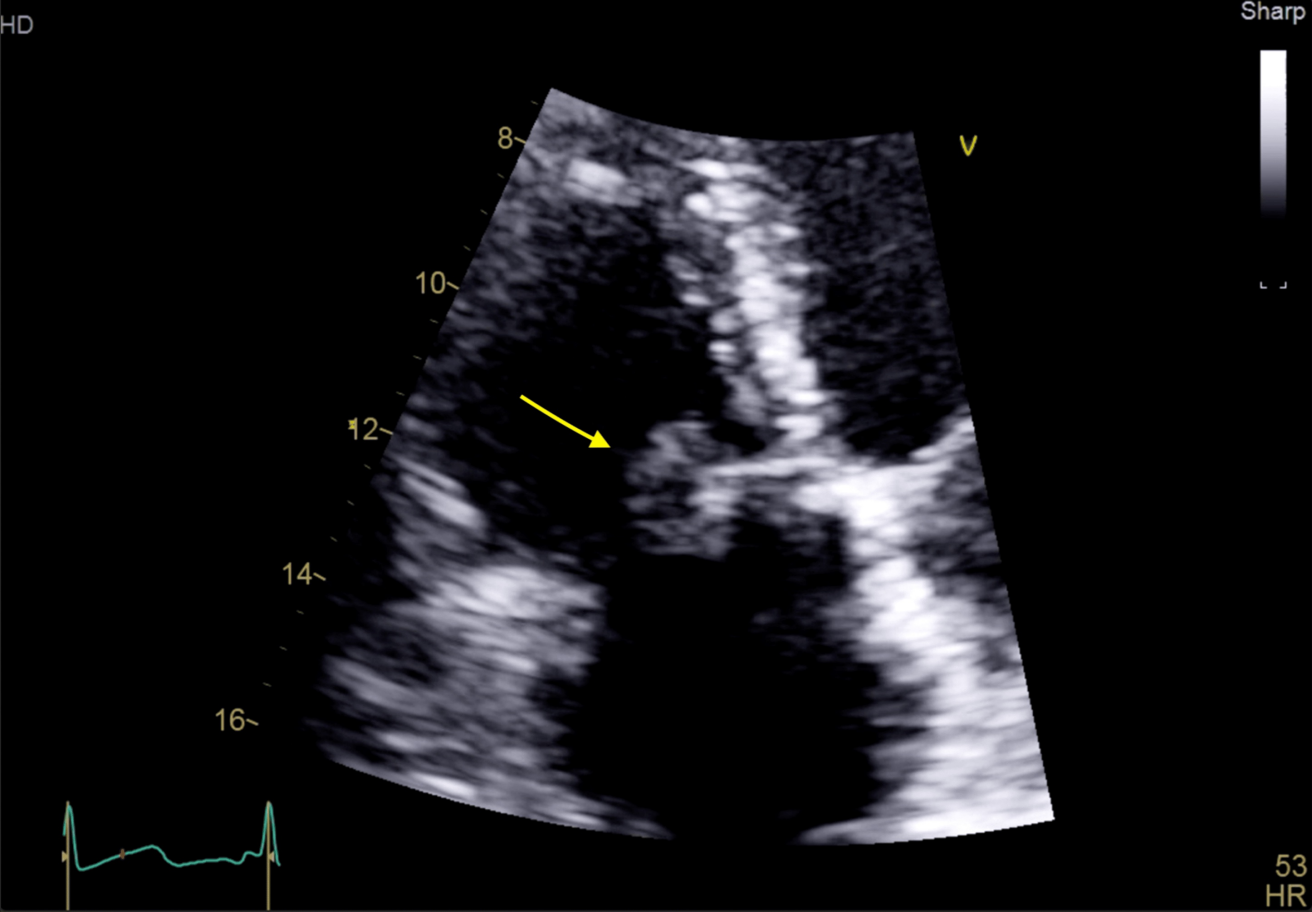
Transthoracic echocardiogram (apical four-chamber view) demonstrating a large mobile mass on the tricuspid valve. The arrow annotation highlights high-density mass on the anterior leaflet of the TV. TV: tricuspid valve

**Figure 3:**
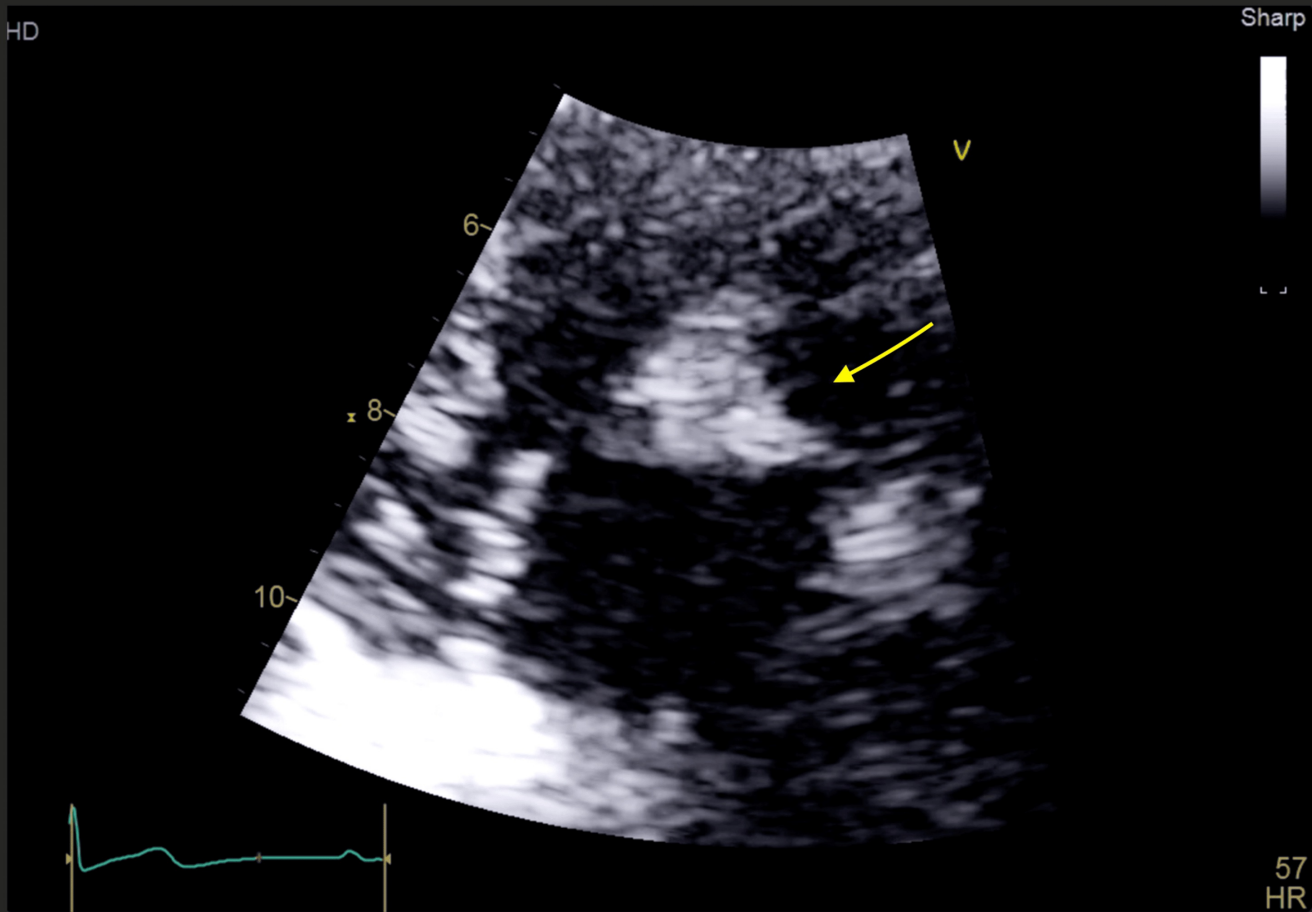
Transthoracic echocardiogram (parasternal long-axis view) demonstrating a large tricuspid mass. The arrow annotation demonstrates a large tricuspid mass within the right ventricular cavity.

**Figure 4:**
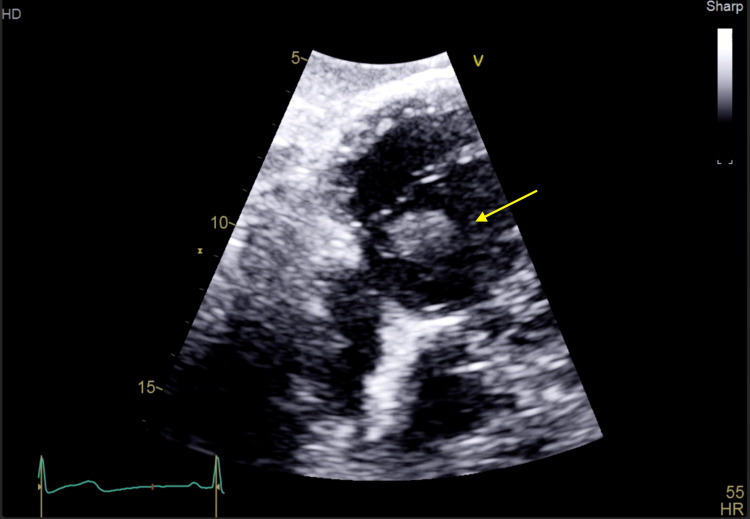
Transthoracic echocardiogram (subcostal view) demonstrating a tricuspid valve mass herniating into the right ventricular cavity. The arrow annotation demonstrates a tricuspid valve mass herniating into the right ventricular cavity.

Transthoracic echocardiography revealed a large mobile vegetation (1.3×1.7 cm) on the anterior leaflet of the tricuspid valve with mild regurgitation and preserved ventricular function (Figures 2-4). The vegetation was seen to prolapse across the tricuspid valve (TV) into the right atrium (RA). Transesophageal echocardiography, including contrast study, excluded a patent foramen ovale, intracardiac shunt, or left-sided valvular disease. Of note, an agitated saline echocardiogram was not performed.

**Figure 5:**
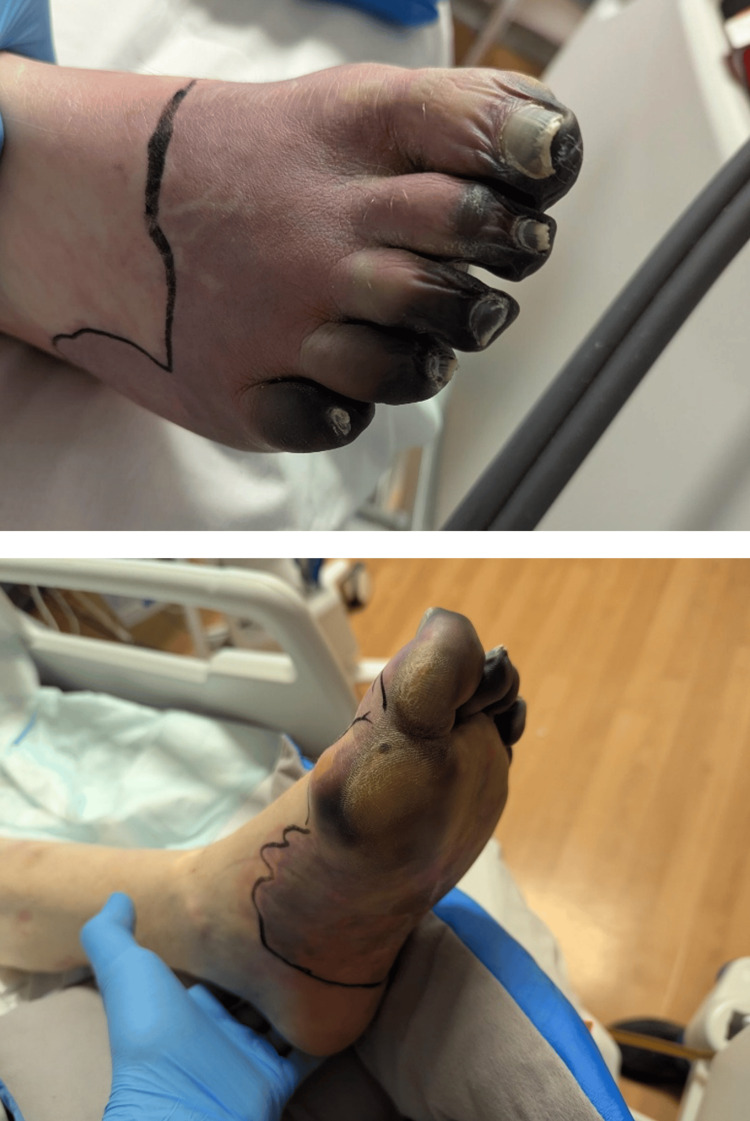
Images taken of bilateral peripheral lower limb ischemic lesions.

The patient received intravenous flucloxacillin and linezolid, continuous renal replacement therapy, vasopressor support, and intravenous immunoglobulin for suspected staphylococcal toxic shock syndrome. He was managed medically without surgery, due to the rapid resolution of his shock state and reduction in vegetation size. After 66 days of hospitalization, he was discharged with residual renal impairment requiring dialysis and dry gangrene of both feet, necessitating bilateral forefoot amputation (Figure 5). Follow-up imaging three months later demonstrated a reduction in the size of the tricuspid vegetation to 1.0x0.9 cm. The cerebral abscess also reduced in size from 20x14x17 mm to 20x13x16 mm on repeat imaging three months later. However, there was a slight increase in surrounding vasogenic edema and mass effect. Given the lack of symptomatology, the patient remained on medical expectant management without neurosurgical intervention.

## Discussion

Infective endocarditis (IE) is an uncommon but serious infection, with an incidence of three to nine cases per 100,000 people annually in developed nations [1]. Despite advances in diagnosis and therapy, IE remains associated with high morbidity and mortality [2].

Uniqueness of the case

Systemic embolization originating from right-sided IE without demonstrable shunting is exceptionally uncommon, with only isolated cases reported. The simultaneous involvement of pulmonary, cerebral, and peripheral vasculature highlights an unusual embolic pattern.

This case illustrates an atypical presentation of right-sided IE. Systemic embolization is classically associated with left-sided lesions, whereas right-sided IE typically results in septic pulmonary emboli [3-5]. The paradoxical embolic pattern in this case occurred despite the absence of an intracardiac shunt or left-sided involvement [6-8].

Potential mechanisms

Several theories have been proposed, including transient right-to-left shunting due to elevated right-sided pressures, pulmonary arteriovenous micro-shunts too small for CT detection, septic microembolization with endothelial disruption (allowing bacteria to traverse pulmonary capillary beds), and undetected pulmonary micro-fistulas.

IE commonly affects structurally abnormal hearts; up to 75% of patients have pre-existing valvular disease [2]. *S. aureus *is the most frequent causative pathogen and carries the highest risk for embolic and metastatic complications [2]. Neurological manifestations, such as abscess or infarction, are more often associated with left-sided disease, especially when mitral valve involvement is present [3,4].

“Echo-negative” endocarditis has been reported in up to 13% of cases, potentially accounting for systemic seeding [5,6]. Pulmonary complications such as empyema and septic emboli are well described in right-sided IE [7], but systemic embolization remains a rare and poorly defined phenomenon [8]. Siddique et al. described a similar presentation involving transient right-to-left shunting [9].

This case reinforces that paradoxical embolization can occur in isolated right-sided IE without a visible intracardiac or intrapulmonary shunt. Clinicians should maintain a high index of suspicion for IE in patients presenting with sepsis and multi-system embolic findings, even when echocardiography suggests right-sided lesions only [1,6].

Virulence of *Staphylococcus aureus*

*S. aureus *is particularly known for atypical metastatic spread through multiple proposed mechanisms, including coagulase-mediated fibrin shielding, adhesins enabling vegetative and endothelial adherence, cytotoxins promoting abscess formation, biofilm formation supporting persistence, and endothelial invasion facilitating dissemination. These features may explain the organism’s uniquely aggressive embolic potential and the features seen in this case.

Differential diagnosis: NBTE

NBTE was considered but deemed unlikely due to persistent *S. aureus* bacteremia and the infectious morphology of imaged vegetations. There was also an absence of underlying malignancy, autoimmune disease, or hypercoagulability [10].

Impact of social determinants of health

The patient’s untreated dental infection and delayed care reflect structural barriers associated with severe infection risk. Recent evidence links socioeconomic inequities to worsened cardiovascular infectious outcomes [11]. Clinicians should maintain a high index of suspicion for IE in patients presenting with sepsis and multi-system embolic findings, even when echocardiography suggests right-sided lesions only. Early MRI was essential for identifying neurological involvement and influencing prompt management. Multidisciplinary management involving cardiology, cardiothoracic surgery, and critical care teams is essential for optimal outcomes. Comprehensive echocardiographic and cross-sectional imaging is crucial to exclude hidden shunts or left-sided disease. This should include shunt-focused imaging techniques such as agitated saline echocardiography. Registry-level studies or prospective cohorts are needed to better understand the incidence and mechanisms of paradoxical embolization from right-sided IE.

## Conclusions

This case highlights a rare presentation of *Staphylococcus aureus *tricuspid valve infective endocarditis resulting in both pulmonary and systemic embolic complications despite the absence of a demonstrable intracardiac or intrapulmonary shunt. Comprehensive imaging, including contrast echocardiography, together with early multidisciplinary management, is critical for improving outcomes. Given the rarity of such presentations, larger studies and registries may help clarify underlying mechanisms and guide management.
